# Thymoquinone alleviates the adverse effects of cyclophosphamide on oogenesis and in vitro fertilization (IVF) in NMRI mice 

**DOI:** 10.22038/ajp.2025.25734

**Published:** 2025

**Authors:** Sima Etebari, Vajihe Rouki, Mohsen Asouri, Mehrab Nasirikenari, Seyed Elyas Meshkani, Mohammad Reza Momtaz, Sakineh Amoueian, Yahya Babaki, Fateme Hajati, Ali Asghar Ahmadi

**Affiliations:** 1 *Department of Physiology, Faculty of Medicine, Sabzevar University of Medical Sciences, Sabzevar, Iran*; 2 *Department of Biology, Faculty of Science, Ferdowsi University of Mashhad, Mashhad, Iran*; 3 *Department of Paramedicine, Amol School of Paramedicine, Mazandaran University of Medical Science, Sari, Iran*; 4 *North Research Center, Pasteur Institute of Iran, Amol, Iran*; 5 *Cellular and Molecular Research Center, Sabzevar University of Medical Sciences, Sabzevar, Iran*; 6 *Pathology Department-Imam Reza Hospital-Mashhad University of Sciences, Iran*; 7 *Mahdiyeh Medical Center, Shahid Beheshti University of Medical Sciences, Tehran, Iran*

**Keywords:** Thymoquinone, Oocyte quality, Cyclophosphamide, Fertilized embryos

## Abstract

**Objective:**

Patients undergoing treatment with antineoplastic drugs face numerous challenges. This study investigates the effects of thymoquinone (Tq) in NMRI mice treated with cyclophosphamide (Cph), aiming to enhance patients' optimism for the future by redirecting their focus towards achieving a normal life and restoring fertility after treatment.

**Materials and Methods:**

Female NMRI mice were divided into five groups: Control, Sham, Cph-treated (CPH), and two groups treated with Tq alongside Cph (TQ5 and TQ10). All mice were sacrificed to aspirate their oocytes for further analysis. The development of embryos up to the blastocyst stage was assessed using in vitro fertilization (IVF) with mature oocytes.

**Results:**

The Tq treatment groups exhibited a dose-dependent decrease in oocyte degeneration compared to the CPH group. A dose-dependent reduction in the rate of metaphase I maturation arrest was also observed in the TQ groups compared to CPH. Tq significantly increased the number of two-cell and four-cell embryos at 24- and 48-hrs post-fertilization compared to the CPH group. Additionally, Tq treatment resulted in significant dose-dependent increases in catalase levels, while malondialdehyde and nitric oxide levels were significantly lower in the TQ groups compared to the CPH groups. Tq-treated groups also demonstrated significant dose-dependent increases in the expression levels of *BMP15* and *GDF9* compared to the CPH group.

**Conclusion:**

In this study, Tq mitigated oocyte factors expression (*GDF9* and *BMP15*) and oxidative damage in ovarian tissues following CPH-induced oocyte degeneration in mice.

## Introduction

As advancements in chemotherapeutic treatments continue, the prognosis for cancer survival is improving. However, chemotherapy and radiation can cause premature ovarian failure in women, leading to infertility (Guida et al., 2016). Women and girls who undergo chemotherapy for common cancers may find themselves unable to conceive or may experience early menopause due to a depletion of their ovarian reserve (Nguyen et al., 2019). The preservation of a female's fertility potential is particularly critical in the ovary, where meiotically arrested, immature oocytes are encased in a single layer of pre-granulosa cells, safeguarding reproductive potential. Primordial follicles can remain in a resting state for up to 50 years until activated to enter the growing follicle pool. To maintain the integrity of the female germline during this prolonged quiescent period, strict genomic surveillance is essential. Currently, in vitro fertilization (IVF) with egg or embryo freezing, as well as ovarian tissue cryopreservation, are the only options available for these patients to preserve their reproductive function. In cases of ovarian failure, reactive oxygen species (ROS) can damage ovarian tissue, a process that contributes to follicle depletion (Blumenfeld, 2012, Morgan et al., 2012). 

The selection of a specific chemotherapy regimen is influenced by various factors, including tumor histology, genetic markers, patient performance status, and prior treatment history. Ongoing research continues to explore the optimization of chemotherapy protocols, including combination therapies and the integration of targeted agents, to enhance therapeutic outcomes while minimizing toxicity. One of the harmful chemotherapeutic agents affecting ovarian reserve is cyclophosphamide (Cph), widely used to treat gynecologic cancers. This drug does not exclusively target cells in the active cell cycle; it can also harm quiescent cells as well as those that are rapidly dividing. Despite ongoing debate, the mechanisms by which chemotherapy damages ovarian reserve remain incompletely understood (Sonigo et al., 2019, Bedoschi et al., 2016). However, some researchers have proposed mechanisms explaining the action of this drug. Hanoux and colleagues stated that Cph functions by forming DNA adducts, ultimately leading to DNA double-strand breaks, although it employs different upstream mechanisms (Hanoux et al., 2007). Since primordial follicles are highly sensitive to genotoxic stress, which can adversely affect fertility, and considering the fact that Cph is non-cycle specific, substantial biological evidence indicates that it can deplete the ovarian reserve by directly damaging oocytes in primordial follicles (Hanoux et al., 2007). Kenche and colleagues linked the observed damage to reactive oxygen species (ROS) generated by Cph, which contribute to tissue damage through the production of toxic ROS. This radical compound can interfere with fertility through several mechanisms, including the inhibition of enzymes, causing membrane and DNA damage, and increasing lipid peroxidation (Kenche et al., 2016, Qin et al., 2016). Consequently, exposure to ROS may lead to changes in spindle morphology, abnormal microtubule arrangement, disruption of scaffolding proteins, and alterations in chromosome positioning, all of which can degrade oocyte quality (Khan et al., 2016, Shaeib et al., 2016). Exposure to Cph is associated with infertility, ovarian failure, and premature ovarian failure in females (Jeelani et al., 2017). However, the precise mechanism of infertility resulting from exposure to Cph has yet to be established.

he role of genes in regulating and ensuring the proper functioning of cellular interactions allows for the study of gene expression changes in response to drugs, thereby monitoring their mechanisms of action. Typically, numerous genes are affected upon drug exposure, making it impractical to study all of them; therefore, changes in the most significant genes are usually prioritized. The growth differentiation factor 9 (*GDF9*) gene plays a crucial role in ovarian physiology, particularly in oocyte maturation and in vitro maturation (IVM). *GDF9* is a member of the transforming growth factor-beta (*TGF-β*) superfamily and is primarily expressed in ovarian follicles, specifically in oocytes (Gilchrist et al., 2006). Research has shown that *GDF9* expression is essential for the proper development and maturation of oocytes. It plays a significant role in regulating various biological processes, including folliculogenesis, oocyte growth, and the coordination of cumulus cell expansion. The expression of *GDF9* is tightly regulated throughout different stages of follicular development, with levels increasing as the oocyte matures. This upregulation is critical for the oocyte's ability to resume meiosis and undergo the necessary morphological changes required for successful fertilization (De Los Reyes et al., 2013).

The bone morphogenetic protein 15 (*BMP15*) gene is another crucial factor in regulating ovarian function and oocyte maturation. As a member of the TGF-β superfamily, *BMP15* is primarily expressed in oocytes and plays a significant role in folliculogenesis, oocyte growth, and the regulation of surrounding granulosa cells, which are essential for follicle development and oocyte maturation. Research indicates that *BMP15* exerts paracrine effects on granulosa cells, promoting their proliferation and differentiation. This interaction is vital for maintaining follicular integrity and orchestrating the hormonal environment necessary for oocyte maturation. Notably, *BMP15* expression tends to increase as follicles progress from the primordial to the antral stage, underscoring its significance in the later stages of follicular development. During oocyte maturation, *BMP15* influences the resumption of meiosis and the cytoplasmic maturation of oocytes. It is involved in the signaling pathways that facilitate the coordination of oocyte and cumulus cell functions, thereby contributing to the overall competence of the oocyte for fertilization and subsequent embryonic development (Cajas et al., 2020).

Currently, researchers are seeking compounds that can mitigate the harmful side effects of chemotherapy drugs. Thymoquinone (Tq), the active phytochemical found in Nigella sativa, is primarily recognized for its antioxidant properties, and its benefits have been extensively studied (Eini et al., 2019). Various conditions can lead to oxidative stress, which Tq helps mitigate by scavenging free radicals. Additionally, evidence suggests that Tq enhances the activity of several antioxidants present in the body, including superoxide dismutase, catalase (CAT), glutathione S-transferases, and glutathione. Consequently, Tq modulates the detrimental effects of numerous suboptimal conditions that contribute to oxidative damage and organ dysfunction. Research has demonstrated that Tq influences the *Bax*/*Bcl2* ratio in a dose-dependent manner and exerts anti-apoptotic effects by modulating oxidative stress and altering cellular redox status (Eini et al., 2019). However, the precise molecular mechanisms by which Tq enhances oocyte development during IVM remain unclear, with dosages utilized in various studies varying widely.

Some studies have reported low doses ranging from 2.5 mg/kg/day (Madani et al., 2023) to 10 mg/kg (Karabulut et al., 2021), while others have utilized higher doses of 20-50 mg/kg/day (Farag et al., 2021). Based on a summary of results from other studies, dosages of 5 mg and 10 mg were selected for this study. The duration of the study was determined by the potential adverse effects of Cph, with the aim of minimizing these effects as much as possible, measured in weeks. In several animal studies, treatment durations ranged from 2 to 4 weeks. In our study, considering the results obtained and the established dosages, we designed the treatment duration to be three weeks (21 days). We investigated the protective effects of Tq on oocyte quality during oogenesis and evaluated the quality of embryos from IVF in NMRI mice treated with Cph.

## Materials and Methods

All reagents and materials were provided by Sigma Chemical Corporation (St. Louis, MO, USA).

### Animals

A total of 60 female and 5 male NMRI mice were used as experimental subjects. All animals were sourced from the North Research Center, Pasteur Institute of Iran. The mice were housed in a controlled environment for two weeks, maintained on a 12-hour light/dark cycle, and had ad libitum access to food and water. The animals were randomly divided into 5 groups, each consisting of 12 mice: control group (untreated), sham group (untreated, with injection and gavage intervention using solvents), Cph group (received 120 mg/kg/week for 4 consecutive weeks via intraperitoneal injection), TQ5 group (received 5 mg/kg Tq for 28 consecutive days via gavage), and TQ10 group (received 10 mg/kg Tq for 28 consecutive days via gavage). The animals were sacrificed one day after the last administration. Oocytes were collected via oviduct dissection following superovulation induced by PMSG (7.5 IU/mouse via intraperitoneal injection, administered 48 hours prior to sacrifice). The maturation rates of the oocytes were evaluated morphologically under an inverted microscope (Motic, AE31) after 24 and 48 hours of culture in α-MEM medium. Fresh mouse sperm was used to fertilize intact mouse oocytes in HTF medium (Pishgam Paya Zist, Iran) containing 4 mg/ml BSA. Samples were assessed for fertilization and the number of two- and four-cell embryos after 24 and 48 hours.

### IVF process

#### Oocyte collection

Under a stereomicroscope, the oviducts were dissected using two insulin syringes and placed in Embryocul-MHRM medium without bovine serum albumin (BSA). In the Embryocul-MHRM fertilization medium containing 15% BSA, oocyte-cumulus complexes were harvested from the dissected medium using a Pasteur pipette. Incubation of the oocyte-cumulus complexes was initiated at 37°C and 5% CO2 until sperm preparation began.

### Sperm suspension preparation

Embryocul-MHRM medium was placed in a test tube and incubated at 37°C and 5% CO2 for 1 hour for each male mouse sacrificed for the experiment. The cauda epididymis from both sides was excised and chopped using small scissors. Sperm were added to 50 µl of Embryocul-MHRM medium droplets containing 10 oocytes after being acclimatized to 37°C under 5% CO2 in humidified air for 4-6 hours. A second transfer of oocytes into 30 µl of culture medium (Embryocul-MHRM medium containing 15% BSA) was then conducted.

### Assessment of embryo development

A stereomicroscope (Nikon Corporation, Tokyo, Japan) was used to observe embryo development on days 1, 2, 3, 4, and 5 following IVF. The percentages of embryos that were arrested were determined.

### Quantitative real-time polymerase chain reaction (qRT-PCR)

A mini kit (RNeasy®) provided by Qiagen GmbH, Hilden, Germany, was used to extract total RNA from ovarian tissue. Total RNA was quantified, and its quality was assessed using a NanoDropTM 2000 spectrophotometer (Thermo Fisher Scientific, Inc.). Subsequently, RNAs were reverse-transcribed using the Prime-Script™ RT reagent kit (Takara Holdings, Inc., Kyoto, Japan). Quantitative RT-PCR analysis was performed using Amplicon’s RealQ Plus 2X MasterMix Green-without RoxTM (Stenhuggervej, Denmark). Specific primers for *BMP15*, *GDF9*, and Glyceraldehyde-3-Phosphate Dehydrogenase (*GAPDH)* genes (Table 1) were obtained from Macrogene (Macrogene Co., Seoul, Korea). A real-time PCR system was employed to amplify the cDNA (Roche Applied Science, Pleasanton, CA, USA). Gene expression data were normalized using *GAPDH*, and the relative expression of target genes was analyzed using the 2^-ΔΔCt^ method. Table 1 lists both forward and reverse primer sequences.

### Measurement of oxidative and nitrosative stress markers

Malondialdehyde (MDA), nitric oxide (NO), and catalase (CAT) levels were determined to examine the effects of Tq on the oxidant-antioxidant balance in ovarian tissues (Bargi et al., 2017).

### Statistical analysis

All statistical analyses were conducted using SPSS version 16. Means and standard deviations were calculated. The means of fragmented oocytes, fertilized oocytes, and two-cell, four-cell, and eight-cell embryos, morulae, and blastocysts were compared between groups using Mann-Whitney tests. The results of oxidative stress were compared using one-way ANOVAs and LSD post hoc analyses. Differences were considered significant at p<0.05.

**Table 1:** qPCR primer sequences

***Genes***		***Primers Sequence***	***Tm***	***Amplicon***	***PCR program***
*GapDH*	Forward	AAGAGGGATGCTGCCCTTAC	59.4	120 bp	Denaturation: 94°C Anealing: 57°C Extention: 72°C
	Reverse	ATACGGCCAAATCCGTTCAC	58.6		
*BMP15*	Forward	CAAGGGAGAACCGCACGATTG	61.9	110 bp	Denaturation: 95°C Anealing: 59.5°C Extention: 72°C
	Reverse	AGGAAAGTCCAGGGTCTGTACATG	61.8		
*GDF9*	Forward	AGCAACCAGGTGACAGGAC	59.5	120 bp	Denaturation: 94°C Anealing: 57.5°C Extention: 72°C
	Reverse	AGAGGCAGAGTTGTTCAGAGTG	60.0		

## Results

### Effects of Tq on descriptive indicators of IVF

Oocyte degeneration during IVM was observed to be dose-dependent in the TQ groups. After 24 hours, the degeneration rates were 41.37% for TQ5 and 39.30% for TQ10, compared to 47% in the CPH group. After 48 hours, the rates were 18% for TQ5 and 14.11% for TQ10, while the CPH group showed a degeneration rate of 67% (Table 2). The progression to the embryonic 2-cell and 4-cell stages was significantly higher in the TQ5 and TQ10 groups after both 24 and 48 hours compared to the CPH group (Table 2). Specifically, after 24 hours, 58.63% of the TQ5 group and 60.70% of the TQ10 group reached the 2-cell stage, while only 53% of the CPH group did. After 48 hours, 56.05% of the TQ5 group and 59.26% of the TQ10 group reached the 4-Cell stage, compared to 48% in the CPH group (Table 2).

### Effects of Tq on descriptive indicators of IVM

In total, 79 oocytes in control group were extracted and analyzed, revealing that approximately 12.66% of immature oocytes were degenerated. On average, 8.86% remained in the germinal vesicle (GV) stage, showing no signs of meiosis onset. Additionally, 29.11% progressed to the meiotic stage 1 but were arrested, while 49.37% advanced to stage 2 and matured (Table 3).

For the 96 oocytes of CPH group, about 33.33% of immature oocytes degenerated. An average of 18.75% remained in the GV stage, with no signs of meiosis onset. Furthermore, 28.13% progressed to meiotic stage 1 but were arrested, while 19.79% advanced to stage 2 and matured (Table 3).

In the 96 oocytes of TQ5 group, approximately 25.23% of immature oocytes degenerated. An average of 17.76% remained in the GV stage, showing no signs of meiosis onset. About 21.50% progressed to meiotic stage 1 but were arrested, while 35.51% advanced to stage 2 and matured (Table 3).

For the 109 oocytes of TQ10 group, around 16.51% of immature oocytes degenerated. An average of 11.01% remained in the GV stage, with no signs of meiosis onset. Additionally, 30.28% progressed to meiotic stage I but were arrested, while 42.20% advanced to meiosis II and matured (Table 3).

**Table 2:** Zygote 2 and 4 cell count after fertilization and IVF

	**24 hr**			**48 hr**		
Group	Degeneration (%)	2 Cells (%)	4 Cells (%)	Degeneration (%)	2 Cells (%)	4 Cells (%)
**Control**	30.77	69.23	0	14.81	21.20	64.00
**Sham**	32.10	67.90	0	16.80	21.07	62.13
**CPH**	47.00	53.00	0	67.00	19.00	48.00
**TQ5**	41.37	58.63	0	18.00	25.95	56.05
**TQ10**	39.30	60.70	0	14.11	26.63	59.26

**Table 3:** Outcomes of IVM oocytes

	Control Count (%)	Sham	CPH	TQ5	TQ10
**GV**	14 (8.86)	24 (15.00)	36(18.75)	38(17.76)	24(11.01)
**MI**	46 (29.11)	38 (23.75)	54(28.13)	46(21.50)	66(30.28)
**MII**	78 (49.37)	70(43.75)	38(19.79)	76(35.51)	92(42.20)
**D**	20 (12.66)	28(17.50)	64(33.33)	54(25.23)	36(16.51)

### Effects of Tq on follicles morphology

**Figure 1:**
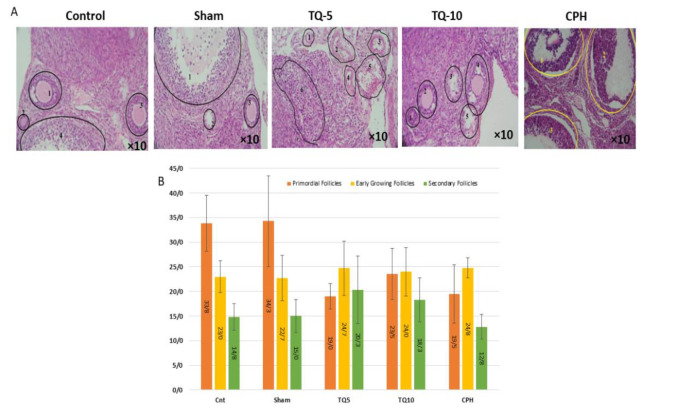
Effects of two doses of Tq on different stages of follicle maturation in ovaries removed from adult female NMRI mice after Cph administration. (A): Photomicrograph of the ovarian section. (B): Average of different stages of follicle maturation in different groups. (Control: 1-Secondary follicle the antrum is not yet integrated; 2- Primary follicle could be seen at higher magnification; 3- Secondary follicle; 4- Antral follicle. Sham: 1- Antral follicle; 2- Vein; 3- Secondary follicle. TQ 5: 1 & 2- Secondary follicles with oocytes forming two small antrums (white corners between granulosa cells); 3- Vein; 4- Antral follicle with pink oocyte, nucleus not visible. TQ 10: 1- Secondary follicle with the left half removed during tissue fixation, pink oocyte; 2 & 4- Secondary follicles with oocyte nucleus not visible; 3 & 5- Large antral follicle with corona radiata surrounding the oocyte, nucleus not visible. CPH: 1, 2, and 3- Large antral follicles, oocyte found in follicle 1.)

Administration of Tq significantly increased the number of primary and secondary follicles across different doses. In contrast, Cph administration led to follicular degeneration (Figure 1A). The CPH group exhibited a decrease in the number of primary and secondary follicles compared to the control group, while both TQ5 and TQ10 groups showed an increase in the number of primary and secondary follicles compared to the CPH group (Figure 1B).

### Effects of Tq on MDA concentrations

**Figure 2:**
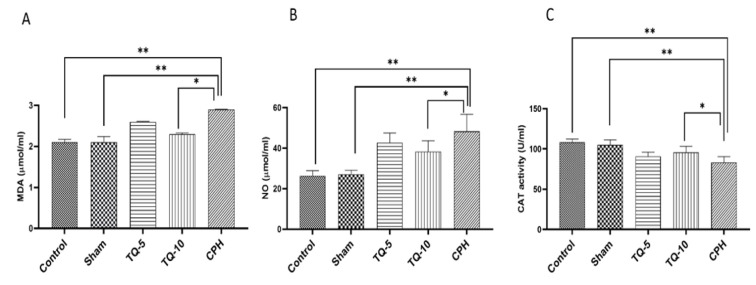
Effects of two doses of Tq on oxidative and nitrosative balance after Cph administration. (A): MDA as an oxidative marker. (B): NO as a nitrosative marker. (C): CAT as an antioxidant marker.

Compared to the control and sham groups, the CPH group exhibited higher MDA concentrations (Figure 2A) (p<0.01). Administration of both doses (5 and 10 mg/kg) of Tq reduced MDA levels compared to the CPH group; however, the lower dose did not have a significant effect (p<0.05 for TQ10; Figure 2A). Therefore, the higher dose was more effective than the lower dose in improving MDA concentration, although TQ5 did not show a significant effect (p>0.05).

### Effects of Tq on NO concentrations

NO metabolites were higher in the Cph-treated group compared to the control and sham groups (p<0.01). Treatment with all Tq doses decreased NO metabolites; however, the TQ5 did not have a significant effect (p<0.05 for TQ10; Figure 2B). No significant differences were observed between the different doses of Tq (Figure 2B).

### Effects of Tq on CAT activity

Administration of Cph also resulted in decreased CAT activity compared to the control and sham groups (p<0.01). Treatment with 5 and 10 mg/kg of Tq improved CAT levels compared to the CPH group, although 5 mg/kg of Tq did not show any significant changes in CAT activity (p<0.05 for TQ10; Figure 2C). Additionally, CAT levels in animals treated with 10 mg/kg Tq were higher than those treated with 5 mg/kg, although this difference was not statistically significant.

### Effects of Tq on gene expression

**Figure 3:**
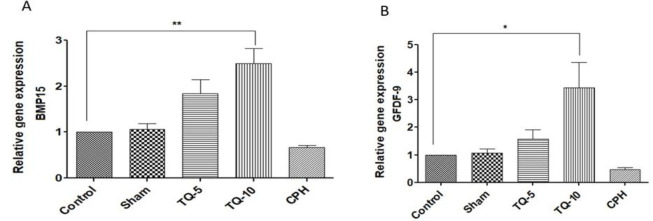
Effects of two doses of Tq on gene expression secreted by oocytes into ovarian follicles after Cph administration. (A): BMP15. (B): GDF9.

Our results demonstrated that Tq administration significantly increased the expression levels of *BMP15* (Figure 3A) and *GDF9* (Figure 3B) compared to the CPH group (p<0.001). Furthermore, *BMP15* and *GDF9* expression levels in the CPH group were lower than those in the control group. While there was a difference between TQ5 and TQ10, it was not statistically significant.

## Discussion

Mammals mature their oocytes by regulating meiotic division, which is closely linked to oocyte maturation. Meiosis begins during embryonic development and concludes at birth, specifically at the diploid meiosis I stage. Following puberty, folliculogenesis resumes with the reinitiation of meiosis at the germinal vesicle (GV) stage, involving the dissolution of the nuclear membrane and the formation of spindle fibers. During nuclear maturation, meiosis resumes, leading to the formation of metaphase II. The aim of the present study was to investigate the effects of Tq on various stages of embryonic development and oocyte maturation. 

Patients undergoing treatment with antineoplastic drugs face numerous challenges. Identifying methods to enhance treatment efficacy, minimize side effects, and improve patient survival rates is a fundamental goal for researchers. This study focuses on Tq, a compound extracted from plant sources known for its beneficial effects on improving fertility in patients. The findings of this study may provide hope for patients by directing their attention towards fertility following treatment.

Despite numerous studies examining the effects of Tq on oxidative stress in various disease models, research on its impact on reproductive cell and tissue disorders, as well as meiosis, remains limited. Specifically, only two studies have 

been conducted on polycystic ovary syndrome (PCOS) models, both reporting favorable effects of Tq on oocytes (Eini et al., 2019, Eini et al., 2020).

After 24 hours, analysis of the oocytes revealed that the treated groups exhibited a higher number of degenerated oocytes compared to the control and sham groups. The TQ5 group experienced a 41.37% loss of oocytes, while the TQ10 group showed a 39.30% loss, and the CPH group had a 47% loss. Tq did not adversely affect oocytes in the GV stage, as indicated by the increased number of oocytes in this stage. The number of oocytes in the metaphase I (MI) stage that resumed meiosis and progressed to maturity was higher in the TQ groups compared to the other groups. Specifically, the TQ10 group had 30.28% of oocytes reaching the MI stage, compared to 29.11% in the control and CPH groups. The present study also demonstrated that the number of oocytes at the metaphase II (MII) stage began to decrease in the presence of TQ5 and TQ10 compared to the control group. The number of two-cell embryos after 24 hours in the treatment groups decreased compared to the controls (69.53%). In terms of Tq's effect on oocyte fertilization, Tq significantly reduced the number of two-cell embryos generated after 24 hours in the treatment groups. After 24 hours, the TQ5 and TQ10 groups had 58.63% and 60.70% of embryos in the two-cell stage, respectively, compared to 53% in the CPH group. A significant reduction in the number of two-cell embryos was observed after 48 hours, with TQ5 (56.05%) and TQ10 (59.56%) showing the highest percentages of embryos reaching this stage, while only 48.8% of embryos in the CPH group did. Based on the results of this study, Tq significantly enhances oocyte maturation, fertilization rates, and the development of two- and four-cell embryos compared to the CPH group.

A study by Eini et al. also reported that the aqueous-alcoholic extract of Nigella Sativa is effective for maturing oocytes in mice with PCOS (Eini et al., 2020). Other research has yielded similar results regarding oocyte maturity and embryo development following the use of various plants. Jefferson et al. demonstrated in their study on the effects of genistein from Glycine max on the ovary that genistein reduces oocyte death when combined with Glycine max, resulting in a greater number of oocytes available for fertilization (Jefferson et al., 2006).

It is crucial for oocytes and embryos to possess both enzymatic and non-enzymatic antioxidants to develop full competency and to prevent or slow the onset of apoptosis (Lian et al., 2013). In the present study, the administration of Tq to oocytes was associated with a decrease in malondialdehyde (MDA) and nitric oxide (NO) levels, along with an induction of catalase (CAT), indicating an improvement in the antioxidant defense system of the oocyte. These results align with those of Busari et al., who showed that Nigella Sativa seed extract and vitamin E improve antioxidative enzyme activity in cells (Busari et al., 2018). Mice treated with Tq after Cph administration exhibited a restoration of antioxidant homeostasis based on the measured antioxidant levels (Alenzi et al., 2010). The extract of Nigella sativa has been reported to activate several antioxidant enzymes, including CAT and glutathione peroxidase, while also scavenging free radicals (Eini et al., 2020). Other studies have demonstrated that Tq, an active component of Nigella sativa, can counteract diethylnitrosamine metabolism induced by oxidative stress by upregulating GST, GPx, and CAT genes in hepatic cells (Eini et al., 2020). A second study clearly showed that Tq administration to PCOS rats resulted in significant improvements in normal follicular development, achieved by suppressing NF-κB signaling, which inhibited *COX2* expression (Arif et al., 2016). Previous research indicated that DHEA overexpresses *COX2* in PCOS mice, potentially leading to differences in cytoplasmic development and the induction of apoptosis (Sancho et al., 2011). In the other study, both control and PCOS mouse oocytes treated with Nigella sativa extract showed decreased *COX2* expression and reduced intracellular ROS production (Eini et al., 2020). Additionally, Arif et al. found that the overexpression of the *COX2* gene and ROS production was inhibited by granulosa cell line exposure to Tq (Arif et al., 2016). Tq's unique ability to suppress ROS production makes it a valuable antioxidant and free radical scavenging due to its distinctive antioxidant activity (Lian et al., 2013).

In recent years, several studies have investigated the pathological processes of primary ovarian insufficiency (POI) concerning two members of the TGF-β superfamily, *BMP15* and *GDF9* (Kuang et al., 2014). *BMP15* and *GDF9* critically influence early folliculogenesis. Both factors stimulate granulosa cell (GC) proliferation in response to FSH activation (Bouali et al., 2016). A dominant heterozygous mutation affecting *BMP15*/*GDF9* can lead to cellular and molecular disturbances, including decreased GC growth and mature proteins, as well as defects in translation and secretion (Bouali et al., 2016, Ziapour et al., 2011). Significant variations in gene dosage can influence ovarian function, resulting in various reproductive issues in females (Persani et al., 2014). *GDF9* and *BMP15* play crucial roles in oocyte development, ovulation, fertilization, and embryonic competence by activating autocrine and paracrine mechanisms during follicular development (Sanfins et al., 2018). Our study found that Tq, compared to the CPH group, increased *BMP15* and *GDF9* gene expression in a dose-dependent manner.

The results indicate that Tq improves maturation, fertilization, and embryonic growth in the two- and four-cell stages, which were adversely affected by Cph administration, by enhancing the growth factors influencing ovarian function and restoring the balance of oxidative stress.
